# Supplementary material for: The effect of ABO blood group and antibody class on the risk of COVID-19 infection and severity of clinical outcomes
Source: Sci Rep. 2021 Mar 11;11:5745. doi: 10.1038/s41598-021-84810-9 (PMC7952683; doi:10.1038/s41598-021-84810-9)
Supplement: Supplementary file 1 — Supplementary Information [file 41598_2021_84810_MOESM1_ESM.docx]

|  | | COV+ICU-  n (%) | COV+ICU+  n (%) |
| --- | --- | --- | --- |
| N | | 2138 | 196 |
| Gender | Males | 642 (30.03) | 157 (80.10) |
|  | Females | 1496 (69.97) | 39 (19.90) |
| Age group | 0-9 | 6 (0.28) | 1 (0.51) |
|  | 10-19 | 28 (1.31) | 0 (0.00) |
|  | 20-29 | 1147 (53.65) | 4 (2.04) |
|  | 30-39 | 788 (36.86) | 17 (8.67) |
|  | 40-49 | 137 (6.41) | 36 (18.37) |
|  | 50-59 | 21 (0.98) | 47 (23.98) |
|  | 60-69 | 6 (0.28) | 49 (25.00) |
|  | 70-79 | 5 (0.23) | 22 (11.22) |
|  | 80+ | 0 (0.00) | 20 (10.20) |
| Nationality | Bahraini | 1891 (88.45) | 102 (52.04) |
|  | Non-Bahraini | 247 (11.55) | 94 (47.96) |

**Supplementary material Table 1**

**Supplementary Table 1.** Demographics (gender, age group and nationality) amongst both the COV+ICU- and COV+ICU+ samples.
